# A single center study that evaluates the preclinical use of a newly developed software and moving bed system to facilitate the spontaneous excretion of residual fragments after primary stone treatment (RIRS or PCNL)

**DOI:** 10.1007/s00345-021-03863-7

**Published:** 2021-10-23

**Authors:** Tao Yang, Rijin Song, Xianghu Meng, Hanping Wei, Xinying Jiang, Xiaoliang Yuan, Xiaowu Liu, Zhimin Jiao, Jun Liu, Honglei Shi

**Affiliations:** 1 Department of Urology, Wujin Hospital Affiliated with Jiangsu University, Changzhou, 213004 China; 2 Department of Urology, The Wujin Clinical College of Xuzhou Medical University, Changzhou, 213004 China; 3 Department of Urology, The First Affiliated Hospital of Nanjing Medical University, Nanjing, 210029 China; 4 Department of Urology, The 940th Hospital of PLA Joint Logistics Support Force, Lanzhou, 730050 China

**Keywords:** Residual fragment, Active lithecbole, Postural drainage, Urolithiasis, Individualized therapy

## Abstract

**Purpose:**

We developed a Postural Drainage Lithotripsy System (PDLS) that uses the patient's computed tomography urography (CTU) data to reconstruct the three-dimensional figure of the renal pelvis, provides an individualized inversion and overturning angle and uses gravity to remove residual fragments (RFs). The purpose of this study was to investigate PDLS in the treatment of renal RFs.

**Methods:**

A stone with a diameter of 4.0 mm was placed in the upper, middle, and lower calyx of the renal model. A total of 60 trials were applied to 20 renal models. The movement trajectory, passage rate, and postural drainage angle of calculi during the treatment of PDLS were observed.

**Results:**

All of the stones in 60 trials were observed to move during treatment, and 53/60 (88%) were relocated successfully to the renal pelvis. The passage rate of the upper calyx was 14/20 (70%), that of the middle calyx was 20/20 (100%), and that of the lower calyx was 19/20 (95%).

**Conclusions:**

PDLS can provide individualized inversion and reversal angles and remove stones from the renal model. More clinical trials are needed to verify the above view and evaluate its efficacy.

**Supplementary Information:**

The online version contains supplementary material available at 10.1007/s00345-021-03863-7.

## Introduction

The management of residual fragments (RFs) after minimally invasive surgery for urinary calculi is controversial in the clinic because these RFs may become high-risk factors, leading to renal colic, obstruction, infection and stone recurrence, for example [1]. At present, percussion, diuresis, and inversion (PDI) are the main methods to remove RFs. Due to the discrepant inverted angle of PDI therapy, its clinical effect is unreliable [2, 3]. We developed a Postural Drainage Lithotripsy System (PDLS) that uses the patient's computed tomography urography (CTU) data to provide an individualized inversion and overturning angle and uses gravity to remove the RFs. Therefore, we designed an in vitro kidney model experiment to verify the feasibility of PDLS.

## Materials and methods

### Postural drainage lithotripsy system

#### Space rotating bed

**Fig. 1:**
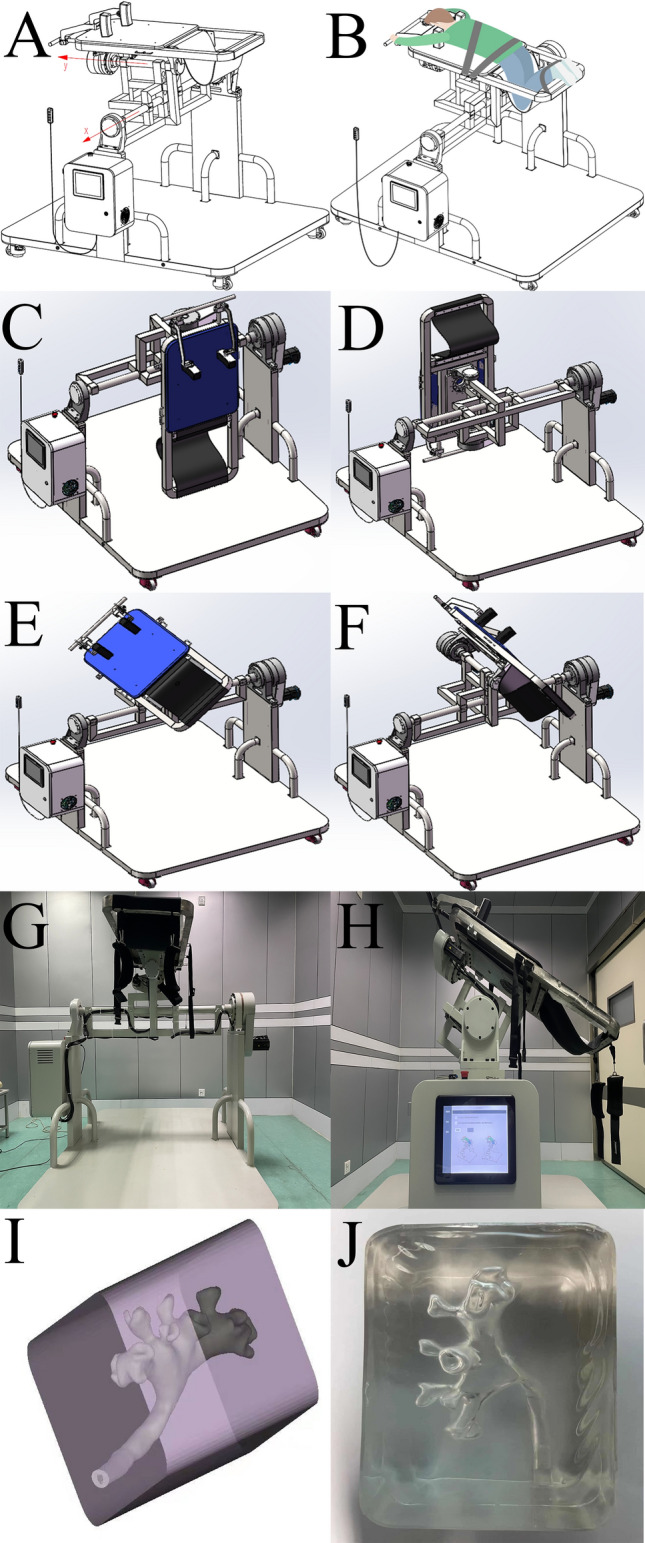
**A** The *X*- and *Y*-axes of the space rotating bed, which are horizontal (0°, 0°); **B** The kidney model is placed on the bed in a prone position; **C** Rotation about the *X*-axis + 90° (toward the standing position), **D** Rotation about the *X*-axis − 90° (toward the inverted position), **E** Rotation about the *Y*-axis − 60° (overturn toward the left); **F** Rotation + 60° about the *Y*-axis (overturn toward the right); **G**–**H** The prototype machine; **I**–**J** Using CTU data of real patients to create a transparent silicone renal pelvis model by 3D printing technology

We applied the novel device spatial rotating bed (manufactured by HIRG International Institute for Research & Innovation) in our study (Fig. 1). The working mechanism of the equipment is to realize the change in the space angle of the bed through rotation around the *X*-axis and *Y*-axis so that the body position of the patient lying prone on the bed is changed accordingly. Rotation range around the *X*-axis is − 90° to + 90°, where ‘ + ’ means toward the standing position and ‘−’ indicates the inverted position; rotation range around the *Y*-axis is − 60° to + 60°, where ‘ + ’ means overturning to the right and ‘−’ means overturning on the left. These rotations occur simultaneously.

#### Stone removal path planning software based on three-dimensional (3D) visualization reconstruction technology

**Fig. 2:**
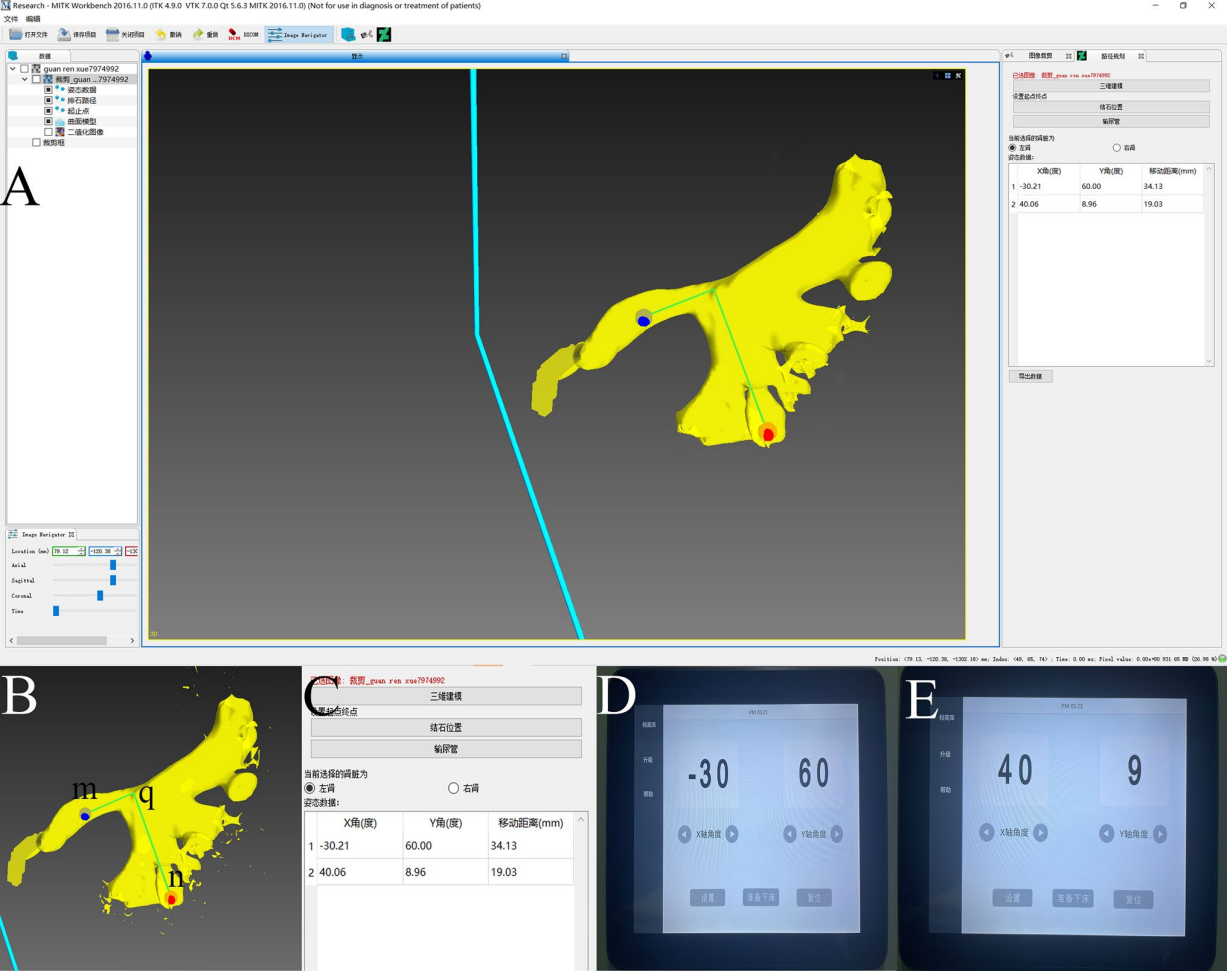
**A** Software operation diagram. The 3D renal pelvis model figure was generated after importing CTU images. **B** *Point n* was set as the stone site, and *Point m* was set as the ureter site. **C** The software generates a green line (stone removal path) and gives the corresponding angle parameters, in which the *n*–*q* segment (*X*, *Y*):(− 30°, 60°), *q*–*m* segment (*X*, *Y*):(40°, 9°). **D**–**E** Sequentially inputting the above parameters on the space rotating bed

Stone removal path planning software is a type of visual assistant software to help doctors find the best postural drainage angle for patients to remove stones (Fig. 2). By importing the CTU data of the kidney, the software can reconstruct the 3D figure of the renal pelvis. In this study, the doctor located the stone on the 3D figure, and stone export was set at the ureteropelvic junction. The stone excretion path was calculated by the software and transformed into the *X*- and *Y*-axis parameters of the spatial rotating bed, and several groups of parameters were given according to the number of paths (Fig. 2). Using these angle parameters, the space rotating bed will change the patient’s position. In the ideal position, the direction of gravity was consistent with the direction of the calyceal outlet, and the stones in the kidney were excreted automatically under the action of gravity.

#### 3D printed kidney model

CTU data of real patients were used to create a transparent silicone renal pelvis model by 3D printing technology (by Hangzhou Regenovo Biotechnology Co., Ltd. Manufacturing). The model truly restored the anatomical structure of the renal collecting system (Fig. 1). During modification, the ureter and bladder were removed, and the urine was sealed in the kidney model. In this study, a total of 20 patients’ CTU data were used to create 20 renal models. The small stone of the kidney was sent to the renal calyx through the flexible ureteroscope.

#### Procedures

A stone with a diameter of 4.0 mm was placed in the upper, middle, and lower calyx of the renal model. One stone was placed in only one calyx, and there was only one stone in each trial. A total of 60 trials were applied to 20 renal models, and each model was utilized in three trials. The model was lying prone on the bed. The doctor imported the corresponding CTU through the above software and calculated the rotation parameters (*X*, *Y*) according to the location of the stone (Fig. 2). Every time the model was changed, the CTU was changed accordingly. The specified rotation parameters (*X*, *Y*) were entered on the control panel of the space rotating bed. After treatment, the renal model was restored to a standing position. The stone was recorded as passed if it moved from a starting location in a renal calyx into the trap at the level of the ureteropelvic junction. The movement trajectory, passage rate, and postural drainage angle of calculi during the treatment of PDLS were observed (Video 1). The small residual renal stones used in this study were removed during retrograde intrarenal surgery (RIRS).

## Results

All of the stones in 60 trials were observed to move during treatment, and 53/60 (88%) were relocated successfully to the renal pelvis. During the treatment of 60 stones, *Y*-axis overturning was > 40° in 46/60 cases and > 50° in 35/60 cases; 7/60 cases needed only one group of angles (one path represents one group of postural angles), 4/60 cases needed three groups of angles, and the remaining 49/60 cases needed two groups of angles (Tables 1, 2).

**Table 1:** Passage rate and angle of the postural drainage machine (*X*°, *Y*°)

Model number	Angle (X°, Y°)			
	Upper calyx	Middle calyx	Lower calyx	Passage rate
Right kidney				
1	(8, 46), (53,0)	(15, 47), (40,0)	(− 63, 60), (43,0)	3/3
2	(54,0)	(58, 60), (35,0)	(− 56, 60), (− 38, 60), (68,0)	3/3
3	(45, 60)	(4, 60), (90, 0)	(− 67, 60), (45, 60)	3/3
4	(90,0), (53, 60)*	(38, 60), (87, 60)	(− 34, 60), (48, 60)	2/3
5	(11, 20), (73, 60)	(44, 60), (− 13, 57)	(− 90,0), (− 14, 60)	3/3
6	(16, 22), (53, 60)	(14, 60)	(− 40, 60), (23,0)	3/3
Left kidney				
7	(11, 22), (35,52)	(24,60)	(− 88,0), (90,60)#	3/3
8	(25,60), (23,16)	(− 1,18), (47,60)	(− 45,15), (67,50)	3/3
9	(15, 47), (40,0)	(− 31,15), (23,33)	(− 59,33), (41,24)	3/3
10	(− 26,18), (66,33)	(24,36)	(− 62,17), (44,21)	3/3
11	(45,40), (23,5)	(32,11)	(− 44,0), (20,57)	3/3
12	(85,0), (18,19)*	(− 31,37), (28,35)	(− 31,37), (28,35)	2/3
13	(− 4,0), (45,0), (30,60)	(17,17), (30,60)	(− 58,36), (60,60	3/3
14	(85,0), (18,19)*	(− 25,60), (0,60)	(− 70,0), (− 5,60)*	2/3
15	(39, 22), (38,27)*	(10,40), (60,48)	(− 45,0), (− 51,60), (67,60)	2/3
16	(24, 22), (38,27)	(− 5,11), (53,23)	(− 45,56), (90,0)	3/3
17	(66, 30), (5,40)*	(21,48), (90,0)	(− 33, 60), (− 27,60), (73,0)	2/3
18	(38,35)	(− 3,58), (60,48)	(− 90,0), (50,55)#	3/3
19	(67, 44), (− 22,36), (55,23)*	(2,23), (57,51)	(− 39), (56,60)	2/3
20	(2,0), (51,48)	(16,38), (64,60)	(− 58,60), (31,36)	3/3
Total	14/20(70)	20/20(100)	19/20(95)	53/60

One group (*X*, *Y*) represents one segment of the path

*Indicates that no successful excretion occurred, and # indicates successful excretion with movement into other kidney calyces. Data are presented as no. passed/no. of attempts (%)

**Table 2:**
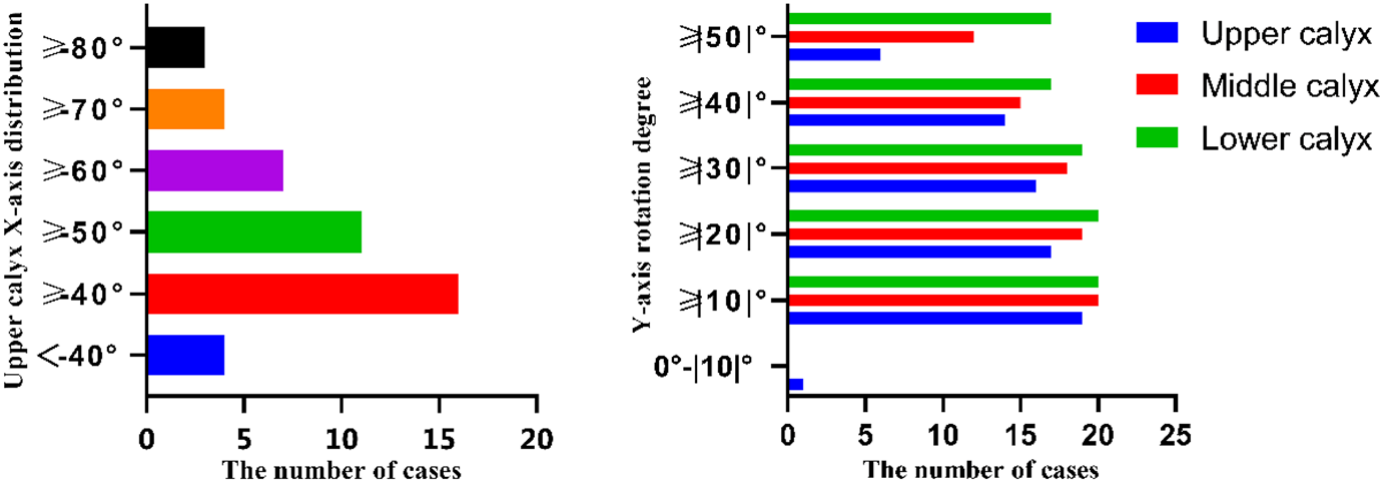
A: Distribution of the X-axis angle in the treatment of lower calyceal calculi. B: Y-axis rotation angle distribution

### Upper calyx

The passage rate of upper calyx stones was 14/20 (70%). During the treatment, only 2/20 cases of the *X*-axis had a negative value (‘−’ means the inverted position): 1 case was − 26° and 1 case was − 4°. In addition, the *Y*-axes of 16/20 cases were ≥ 30°, 14/20 cases were ≥ 40° and 6/20 were ≥ 50°. Only one group of angles was needed in 3/20 cases, three groups of angles were needed in 1/20 cases, and two groups of angles were needed in 16/20 cases.

### Middle calyx

The passage rate of the middle calyx was 20/20 (100%). Only 7/20 cases of the *X*-axis had a negative value, and the angle of inversion in 2/20 cases was more than − 30°. In addition, there were 18/20 cases with *Y*-axis overturning ≥ 30°, 15/20 cases ≥ 40°, and 12/20 cases ≥ 50°. A total of 4/20 cases needed only one group of angles, and 16/20 cases needed two groups of angles.

### Lower calyx

The passage rate of the lower calyx was 19/20 (95%). It was also observed that in 2 of 20 cases lower calyx stones first fell into the upper calyx and then passed through the ureteropelvic junction, and that in only 1 of 20 cases did lower calyx stones fall into the middle calyx and pass through. The *X*-axis was negative in all 20 cases, of which 16/20 cases were greater than − 40°, 11/20 cases were greater than -50°, 7/20 cases were greater than − 60°, 4/20 cases were greater than − 70°, and 3/20 cases were greater than − 80°. In addition, 19/20 cases had *Y*-axis overturning ≥ 30°, and 17/20 cases had *Y*-axis overturning ≥ 50°. Three groups of angles were needed in 3/20 cases, and two groups of angles were needed in 17/20 cases.

## Discussion

The lifetime risk of urolithiasis is 6–12%, and the 10-year recurrence rate is as high as 50% [4]. Minimally invasive surgery, such as RIRS and percutaneous nephrolithotomy (PCNL), is the gold standard for the treatment of urolithiasis in modern urology [5]. However, RFs after minimally invasive surgery are an inevitable problem. Traditionally, nonobstructive RFs with diameter ≤4 mm was called “clinically insignificant residual fragments (CIRFs)” because of their higher spontaneous excretion rate and lower reintervention rate. However, within 2 years after minimally invasive surgery, 26% of the patients with CIRFs developed the above symptoms and needed secondary intervention [6]. Repeated surgery and treatment not only reduce the quality of life of patients but also increase medical costs [7]. Therefore, it is very important for the health of patients to ensure the complete removal of stones. At present, PDI are the main methods to remove RFs. Although PDI therapy has been demonstrated to be safe and effective and has been used in clinical practice, it has drawbacks. The angle of inversion is discrepant, varying from 12° to 45° to even greater than 60°–70°, and there is no single credible value. Therefore, it is necessary to explore a more effective and reasonable degree of inversion [3]. The innovation of the system we developed is that it can guide doctors to implement individualized inversion and overturning angles according to the CTU data of different patients and make full use of gravity to excrete RFs.

The diameter of the stones used in our experiment was 4.0 mm because stones > 4 mm were usually dusted by the laser or removed through the stone basket during the operation. If an operation is to be defined as successful, the diameter of RFs should be ≤ 4 mm. When the CIRFs of 4 mm can be removed, stones < 4 mm should also be removed.

In clinical practice, renal stones are excreted preferentially from the upper calyx, and small stones located in the lower calyx may be difficult to move into the renal pelvis. In our experiment, the stone passage rate of the lower calyx was higher than that of the upper calyx (19/20 vs. 14/20). Because RFs primarily remain in the lower calyx, we did not study the reason for the low passage rate of the upper calyx.

In the course of the study, it was also found that the *X*-axis of the lower calyx stone was often more than − 50° during the treatment and even greater than − 70° in some cases. A greater negative value of the *X*-axis means a greater degree of inversion (Table 2). If the treatment target is the patient, the degree of tolerance to inversion and reversal needs to be further studied. In the treatment of upper and middle calyceal stones, the degree of *X*-axis inversion is low or not inverted. Therefore, we did not discuss the *X*-axis angle at this time. Although 46/60 cases of *Y*-axis overturning are > 40° and 35/60 cases are > 50°, we thought that left and right overturning would not significantly reduce patient comfort, but further experiments were needed to study the relationship between passage rate, comfort and rotation angle, as well as whether a satisfactory passage rate and comfort can be obtained after lowering a certain inverted and overturning angle.

In total, 49/60 (82%) cases needed two groups of angles because most of them needed two pathways: renal calyx–renal pelvis and renal pelvis–ureter, and more pathways and postural angles may be needed when the anatomy of the renal pelvis and calyx is complex.

The transparent silicone kidney model made by 3D printing technology based on the CTU images of real patients can be used as a substitute for applying PDLS to treat RFs. The transparent nature of the model enables clear observation of the trajectory of stone movement during the experiment. During the experiment, no intervention measures will be applied and the rotation basis of the bed is only determined from the angle calculated by the above software. In addition, the ureter and bladder were removed during modification. Because the purpose of our treatment is to discharge the CIRFs into the UPJ, CIRFs will be treated on the third day after operation when clinical study (which means that CIRF is still small enough during treatment, without increasing in diameter) [8], and the ureteral stent will still be placed in the ureter during treatment, which can dilate the ureter. Therefore, we believe CIRFs can pass through the ureter smoothly.

External physical vibration lithecbole (EPVL) is equipment that can effectively increase stone clearance after extracorporeal shock wave lithotripsy (ESWL) and RIRS without obvious adverse effects [9–11]. The latest EPVL can also provide a certain inverse and overturning angle, but the amplitude is low (inverted angle 0°– ± 35°, overturned angle 0°– ± 20°) [12]. EPVL or ESWL joint PDLS may be more efficient. Ultrasonic propulsion (UP) is a new device and technology that utilizes focused ultrasonic waves to reposition stones in collection systems [13]. The most important aspect for UP is to find the best alignment of the directions of propulsion, gravity, and kidney export. If there is not sufficient power to push the stone from the kidney, the stone may fall back to the original position. Since the PDLS can keep the direction of gravity consistent with the direction of the calyceal outlet, a higher stone clearance may be obtained when combined with EPVL vibration or UP propulsion. More experiments are needed to assess the feasibility of this combination treatment.

Limitations to the current study include the use of a renal model instead of real patients, using only one kidney stone size, and placement of only one stone in each calyx. In addition, we did not study the movement of stones with different shapes. In the next step, we plan to study multisite and multishape stones and place multiple stones of different sizes in each renal calyx.

## Conclusions

Our study demonstrates the feasibility of treating RFs in PDLS, which may have a great impact on the management of small fragments. The advantages of this new technique include individualized postural drainage, applicability to different renal calyces, repeatability, and lack of need for sterilization or anesthesia. More experiments will be conducted to assess the relationship between stone passage, comfort and angle and the feasibility of joining this method with other stone excretion techniques.

## Supplementary Information

Below is the link to the electronic supplementary material.

Supplementary file1 (MP4 239044 KB)

## Abbreviations

RFs: Residual fragments
PDLS: Postural drainage lithotripsy system
CTU: Computed tomography urography
PDI: Percussion, diuresis, and inversion
3D: Three-dimensional
RIRS: Retrograde intrarenal surgery
EPVL: External physical vibration lithecbole
ESWL: Extracorporeal shock wave lithotripsy
UP: Ultrasonic propulsion
